# Beyond genes‐for‐behaviour: The potential for genomics to resolve long‐standing questions in avian brood parasitism

**DOI:** 10.1002/ece3.70335

**Published:** 2024-11-17

**Authors:** Katja Rönkä, Fabrice Eroukhmanoff, Jonna Kulmuni, Pierre Nouhaud, Rose Thorogood

**Affiliations:** ^1^ HiLIFE Helsinki Institute of Life Sciences University of Helsinki Helsinki Finland; ^2^ Research Programme in Organismal & Evolutionary Biology, Faculty of Biological and Environmental Sciences University of Helsinki Helsinki Finland; ^3^ Centre for Ecological and Evolutionary Synthesis, Department of Biology University of Oslo Oslo Norway; ^4^ Department of Evolution and Population Biology, Institute for Biodiversity and Ecosystem Dynamics University of Amsterdam Amsterdam The Netherlands; ^5^ CBGP, INRAE, CIRAD, IRD, Montpellier SupAgro Univ Montpellier Montpellier France

**Keywords:** avian brood parasitism, behavioural ecology, behavioural genomics, landscape genomics, population genomics, Tinbergen's four questions

## Abstract

Behavioural ecology by definition of its founding ‘Tinbergian framework’ is an integrative field, however, it lags behind in incorporating genomic methods. ‘Finding the gene/s for a behaviour’ is still rarely feasible or cost‐effective in the wild but as we show here, genomic data can be used to address broader questions. Here we use avian brood parasitism, a model system in behavioural ecology as a case study to highlight how behavioural ecologists could use the full potential of state‐of‐the‐art genomic tools. Brood parasite–host interactions are one of the most easily observable and amenable natural laboratories of antagonistic coevolution, and as such have intrigued evolutionary biologists for decades. Using worked examples, we demonstrate how genomic data can be used to study the causes and mechanisms of (co)evolutionary adaptation and answer three key questions for the field: (i) Where and when should brood parasitism evolve?, (ii) When and how should hosts defend?, and (iii) Will coevolution persist with ecological change? In doing so, we discuss how behavioural and molecular ecologists can collaborate to integrate Tinbergen's questions and achieve the coherent science that he promoted to solve the mysteries of nature.

## Graphical abstract

Reviewing the usage of molecular tools in avian brood parasitism, we noticed a gap in utilizing genomics in behavioural ecology. Here we use the study of avian brood parasitism to discuss how a new integration of molecular and experimental approaches can help resolve three outstanding questions in behavioural ecology. In the spirit of Tinbergen, we encourage collaboration and integration of different tools to promote new advances in behavioural ecology.

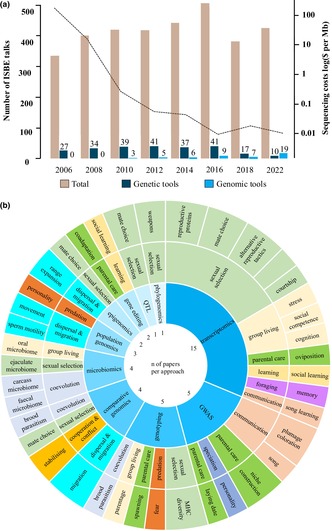

## 1 BEHAVIOURAL ECOLOGY AND GENOMICS

**FIGURE 1:**
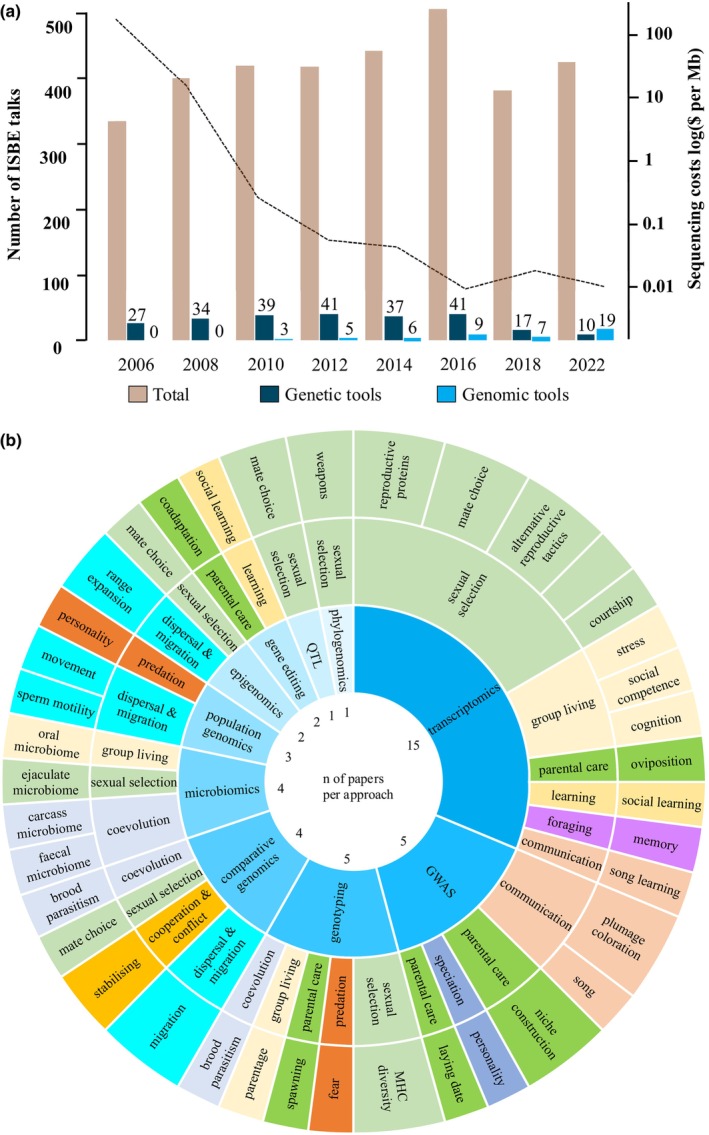
The use of genomic (i.e. next‐generation and third‐generation methods using high‐throughput sequencing) and genetic (any non‐genomic method utilising DNA or RNA) tools in behavioural ecology using (a, b) abstracts from International Society for Behavioural Ecology (ISBE) conference talks. Despite a dramatic reduction in sequencing costs (dashed line), genomic tools are used rarely in behavioural ecology (a), although a range of methods and approaches are applied across a wide range of topics (b). The titles and abstracts of accepted talks in ISBE conferences between 2006 and 2022 were checked manually from abstract booklets for mentioning usage of “genomic methods” (i.e. next generation, high throughput, deep or RAD sequencing, *omics*, transcriptom* or RNA‐sequencing). Talks mentioning usage of molecular methods (or DNA or RNA) that were not genomic were counted as using “genetic methods”. Only regular talks with abstracts in the programs were included, leaving out plenaries, keynote speakers and cancelled talks from ISBE2022 from the total number of accepted talks. Only articles clearly stating the genomic methods used were included to make (b). The included ISBE talks with abstracts are listed in the Table S1.

In 1963, Tinbergen published his landmark paper that has changed the way ecologists and evolutionary biologists study behavioural traits (Bateson & Laland, 2013). He proposed that understanding behaviours as adaptations would require integrating the answers to four complementary questions about (i) the underlying mechanisms of the trait, (ii) its development, (iii) evolutionary history, and (iv) its effects on fitness (Tinbergen, 1963). Genomic data can be used to help address each of these questions but, until recently, has been largely underutilised by behavioural ecologists (Figure 1a). Most work has attempted to answer Tinbergen's question about mechanisms (Bengston et al., 2018; Rittschof & Robinson, 2014). Behaviours, however, are not only traits that evolve; they can affect evolutionary processes by influencing which genomes interact in time and space. For example, natal and breeding dispersal (followed by mate choice) determine gene flow and therefore the starting point for founder effects and strength of drift. Behaviours can also influence heritability (i.e. non‐genetic inheritance and indirect genetic effects, Adrian‐Kalchhauser et al., 2020) and may even be a precursor for genetic adaptation (i.e. Bailey et al., 2017; Jarrett & Kilner, 2017) as behaviour is often a first and fast response to environmental change (Wong & Candolin, 2015). Therefore, here we move beyond mechanisms to highlight how current genomic tools (listed in Table 1) can be used to study the evolutionary history, development, and fitness consequences of behavioural adaptations, and to better incorporate behavioural variation in studies of genetic change.

**TABLE 1:** Genomic tools to study behaviour.

Tool	Data	Definition	Goal	Example studies	Further reading
Genotyping	SNPs from reduced representation approaches (e.g. RAD‐seq)	Determining individual genotypic differences at a subset of loci	Identifying DNA markers for characterising population structure (defining biological populations), parentage, breeding or tracking disease	International HapMap Consortium (2003) and Spurgin et al. (2019) for great tit HapMap projects	Ragoussis (2009)
Quantitative genetics	Whole‐genome sequencing and SNPs from reduced representation approaches	Characterising the statistical association between a phenotypic trait and genotypes (quantitative trait loci [QTL] mapping and genome‐wide association study [GWAS])	Inferring the genetic architecture and heritability of polygenic traits, detecting relevant loci to study molecular mechanisms underlying phenotypes of interest	See Merrill et al. (2019) for a simple oligogenetic basis for mate recognition in *Heliconius* butterflies, Küpper et al. (2016) for a supergene determining behavioural lekking strategies in the ruff *Calidris pugnax*, Ameline et al. (2021) for resistance genes in *Daphnia* against a bacterial pathogen, Wu et al. (2020) for altruisim genes between european earwig *Forficula auricularia* parents and offspring	Gienapp et al. (2017) for QTL mapping, Santure and Garant (2018) for GWAS
Metagenomics	DNA sequencing from environmental or clinical samples	Study of the microbiome, which is the community of micro‐organisms (e.g. bacteria, protozoa, fungi and viruses) inside a given habitat (e.g. body part, organism or environmental sample)	Understanding the factors causing variation in microbiomes and how the microbiome interacts with its environment	Shukla et al. (2018) for burying beetles *Nicrophorus vespilloides* regulating carcass microbiota for their offspring, Matheen et al. (2022) for factors affecting wild bird microbiota	Kumar (2021)
Phylogenomics	Thousands of loci, e.g. ultra conserved elements UCE	Study of evolutionary relationships using phylogenetic inference drawn from genomic data	Testing evolutionary hypotheses either by sequence‐based methods or whole‐genome features	Griesser et al. (2017) for evolution of cooperative breeding in birds. For other examples see Question 1	Adams and Collyer (2018), Cornuault and Sanmartín (2022), Höhna et al. (2016), Kapli et al. (2020), Liu et al. (2019)
Comparative genomics	Whole genome assemblies	Comparisons of the genomes of different taxa (e.g. genome structure, gene content)	Understanding genome evolution at larger evolutionary scales	Dussex et al. (2021) to investigate genes related to tool use in corvids	Mushegian (2010)
Transcriptomics	RNA sequencing	Study of the gene expression (transcript levels) within an organism or tissue under given conditions	Detecting genes and defining regulatory pathways underpinning trait expression	Louder et al. (2020) aggressive host responses to cowbirds	Jax et al. (2018), Larsson et al. (2021), Wang et al. (2009)
Epigenomics	DNA sequencing (e.g. CHIPseq, FAIREseq)	Study of epigenetic processes in the genome, such as methylation and altered expression rates	Understanding variation in behaviour that is not determined by genotype	Lehto and Tinghitella (2020) parent exposure to predation alters offspring mating behaviour in three‐spined sticklebacks *Gasterosteus aculeatus*	Battaglia (2020) for discussion on evolutionary implications and twin studies
Gene editing	CRISPR	Manipulation of the genome by deleting, inserting, or replacing a gene sequence	Producing transgenic individuals to test gene functions	Trible et al. (2017) used CRISPR/CAS9 to knock‐out genes associated with social behaviour in raider ants *Ooceraea biroi*	Cooper et al. (2018), Reardon (2019), Woodcock et al. (2017)
Population genomics	Whole‐genome sequencing and SNPs from reduced representation approaches	Study of evolutionary processes at the population level	Characterising the roles of selection, gene flow, mutation, recombination and genetic drift on the patterns of genetic diversity	Branch et al. (2017) for population structure across elevations in mountain chickadees *Poecile gambeli*	Luikart et al. (2003), Rajora (2019)
Landscape genomics	Whole‐genome sequencing and SNPs from reduced representation approaches	Characterising the statistical association between genotypes and environmental variables	Studying evolutionary mechanisms in a spatial context (e.g., dispersal barriers, local adaptation)	Dudaniec et al. (2018) used Gene–Environment Association analyses (GEA) to detect signs of selection during a range expansion in a damselfly, and Question 3	Rellstab et al. (2015)

*Note*: These include common methods and ‐omics approaches, some of which are already utilised in behavioural ecology (see Figure 1). Here we provide example studies including behavioural traits but not necessarily from behavioural ecology, and suggestions for further reading specific to each tool. Many helpful broad overviews of genomic tools are available (Bild et al., 2014; Kratochwil & Meyer, 2015).

Earlier developments in genetic approaches led to major advances in behavioural ecology (e.g. using microsatellites to determine parentage, Avise, 1996). So why have calls to use genomic tools (Bengston et al., 2018; Rittschof & Robinson, 2014; Sorenson & Payne, 2002; Springer et al., 2011) had limited impact on the field (Figure 1)? This might be because much of the emphasis has been on ‘finding the gene/s for a behaviour’ but this is still rarely feasible or cost‐effective in the wild (Bubac et al., 2020) (See Box 1). Behavioural and molecular ecology have also historically relied on different approaches with increasingly divergent jargon. While both behavioural and molecular ecologists study adaptation and address ultimate ‘why’ and proximate ‘how’ questions, behavioural ecology was founded on the ‘phenotypic gambit’, where knowing heritable genetic mechanisms is considered unnecessary as long as the trait correlates with fitness proxies (Grafen, 1984). Meanwhile, to resolve technological and methodological issues inherent to studying the heritability of polygenic traits (like behaviour), molecular ecologists have largely focused on traits thought easier to measure (e.g. morphology, often colouration; Dochtermann et al., 2019) and assumed to be determined by few, large‐effect loci (Kraus, 2019). However, heritability estimates for behavioural traits (when investigated) are often within range of those for physiological and life‐history traits (Dochtermann et al., 2019) and it is becoming evident that a single gene or supergene is unlikely to be a common explanation for phenotypic variation (Bubac et al., 2020), including in model systems (e.g. chicken *Gallus gallus* plumage and egg colouration: Vignal & Eory, 2019). Furthermore, with rapid advances in epigenomics and knowledge of regulatory mechanisms, it is becoming clear that the genome is an environmentally responsive entity with analogies to behaviour (Rittschof & Robinson, 2014): how the genome is read and transcribed changes throughout the life of an organism and can transmit environmental responses more quickly across generations than previously thought. Taking the behaviour of the genome into account may even have the potential for a paradigm shift in our understanding of evolution (Corning, 2020).

BOX 1 **Behavioural genomics in the wild**

Many of the previous calls for behavioural ecologists to adopt genomic tools have focused on using approaches from behavioural genomics (Bengston et al., 2018; Fischer et al., 2021; Rittschof & Robinson, 2014; Springer et al., 2011). Here, the goal is to uncover the molecular mechanisms underlying behavioural traits and ideally, this requires measuring levels of gene expression during a behavioural response (within the whole organism, organs or single cells; see transcriptomics in Table 1), determining which genes are up‐ or downregulated compared to when the behaviour is not expressed, then experimentally testing causation by e.g. gene knock‐out experiments or crosses (gene editing in Table 1). Most of the methods available, however, were originally developed using model organisms (e.g. humans *Homo sapiens*, fruit fly *Drosophila melanogaster*, zebra fish *Danio rerio*, laboratory mice *Mus musculus* and rats *Rattus norvegicus*), and much of the success thus far has come from studying organisms that lend themselves to highly controlled laboratory‐based experiments (e.g. great tits *Parus major*, Laine et al., 2016) or where lethal sampling of tissues has fewer ethical concerns (e.g. *Heliconius* butterflies: Rossi et al., 2020). This limits wide‐scale adoption to study behavioural traits in wild populations. For example:

Quantitative trait locus (QTL) analyses designed to account for polygenic heritability require large sample sizes of behaviourally‐phenotyped and pedigreed individuals, coupled with long‐term monitoring, which can be difficult to obtain and require significant resources, including long‐term funding, for organisms in the wild (Bubac et al., 2020).

Top‐down approaches such as genome‐wide association studies (GWAS) are most effective when traits are determined by few genes or ‘supergenes’ and associations are not masked by environmentally induced plasticity (see Table 1 for examples). However, large‐effect loci and simple genetic architectures only occasionally explain phenotypic variation (Bubac et al., 2020) and identifying small effect genes contributing to behavioural traits has proven challenging even in humans, despite hundreds of thousands of genomes sequenced (Abdellaoui & Verweij, 2021). In addition, regardless of the genetic architecture, environmental variation may only affect the presence of alleles in one setting (i.e. conditional neutrality, Anderson et al., 2013), or produce an opposing gene expression pattern in two different environments (i.e. plasticity), meaning that the different phenotypes cancel out the association. GWAS methods are however being developed to account for confounding variation (e.g. naturalGWAS: François & Caye, 2018 and RepeatABLE: Rönnegård et al., 2016).

Bottom‐up gene expression studies depend on the researchers' ability to induce and measure relevant and consistent behavioural responses (including the control or reference behaviour), and determine the correct timing and location to sample tissue where the genes are expected to be expressed (which can be measured within the whole organism, organs or single cells). This is especially problematic when studying behaviour in the wild as there is substantial uncertainty regarding where and when to sample expression of genes and behaviour (Rittschof & Hughes, 2018) and most tissues require lethal sampling. Furthermore, lethal sampling makes it impossible to continue behavioural measurements to probe individual variation and plasticity. Developing and validating methods using blood samples may provide a useful alternative (Anderson et al., 2022).

Furthermore, the lack of relevant functional annotation of genes and knowledge of regulatory gene networks is a major hindrance for any genome‐wide or gene expression studies in the wild (Bengston et al., 2018; Vignal & Eory, 2019), although available annotations for markers expressed (or underexpressed) are increasing, e.g. social behaviour in quail *Coturnix japonica* (Morris et al., 2020).

Finally, gene editing approaches using methods such as CRISPR‐cas9 (Table 1) to experimentally test associations are not feasible (or ethical) with most wild vertebrate study organisms (Lunshof, 2015) and are still largely a blunt tool to probe complex traits.

More broadly, there is ongoing debate as to whether searching for a ‘gene (or genes) for behaviour’ is worthwhile (Abdellaoui & Verweij, 2021; Zuk & Balenger, 2014). Detailed studies on the 3D structures of (human) genomes and epigenomics are revealing that phenotypic traits are often determined by complex regulatory pathways, affecting the timing of expression in networks of tens to hundreds of interacting genes (e.g. epistasis and pleiotropy), and even the formation of supergenes is regulated by several genes associated with hormones (Maney & Küpper, 2022). These developments have led to a paradigm shift in genomics from focusing on gene sequences, to understanding the regulatory and evolutionary mechanisms occurring at the molecular level (Charney, 2012; Corning, 2020; see e.g. Larsson et al., 2021 for insights from single‐cell genomics). In this review, we therefore outline approaches and methods to advance the field of behavioural ecology without the need to ‘find a gene for behaviour’.

Behavioural ecology provides a rich understanding of why behaviour and associated traits (e.g. morphology and physiology) evolve at the phenotypic level, along with a tool‐kit of quantitative and experimental methods to address how the actions of individuals influence population‐level processes in the wild (Cuthill, 2005; Owens, 2006). In contrast, the declining costs of next‐generation sequencing (Figure 1a) have helped molecular ecologists use genomic data across a range of approaches and (non‐model) organisms to identify genes and traits relevant for fitness and adaptation (Table 1, Barrett et al., 2019; Ellegren, 2014; Hancock et al., 2011). For example, (i) increasing the number of genetic markers across the genome can help improve the robustness of phylogenetic trees and comparative analyses, by resolving discrepancies between different loci (Jarvis et al., 2014); (ii) by comparing neutral and selected markers across genomes, we can now infer population demographic histories and local adaptation in addition to population structure and gene flow (Hahn, 2018); and (iii) genome sequencing provides increased resolution to determine parentage and facilitates inferring offspring (or hybrid) fitness (through pedigree reconstruction, e.g. Chen et al., 2016). Perhaps most importantly, (iv) genomics allows us to go further than just improving on previous genetic methods: we can acquire information about species' evolutionary history and adaptive potential and predict population resilience in the face of global change (Bay et al., 2018).

Massive genomic datasets for species where we already have (or have the potential to collect) rich behavioural data are becoming available (e.g. Avianbase: Eöry et al., 2015), so how can we overcome this cultural divide in approaches and inherent jargon (e.g. Table 1)? Here we bring together behavioural and molecular ecologists (Box 2) to work through a case study example and demonstrate how we can combine information from both the genome and behaviour to study adaptation. We use avian brood parasitism, one of the classic textbook examples in behavioural ecology (Thorogood et al., 2019), and assess what is (and what is not) possible with the rapidly expanding, but often overwhelming (Krüger et al., 2017; Travisano & Shaw, 2013; Zuk & Balenger, 2014), a range of genomic tools and analytical methods available to address broad questions of interest.

BOX 2 **Forming collaborative teams to take the field forward?**

**FIGURE B1:**
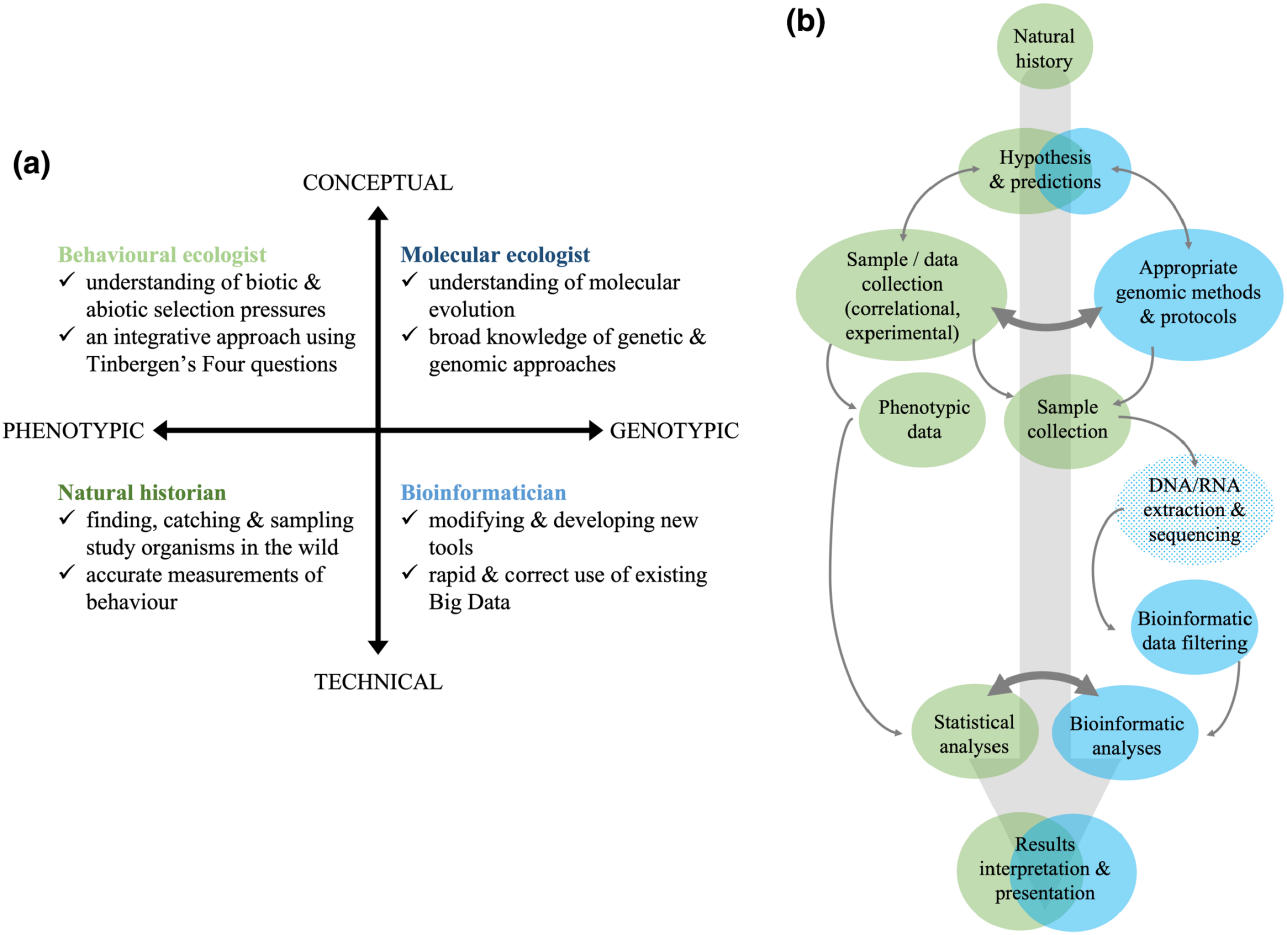
(a) Composition of an ideal collaborative team to bridge the gap between molecular and behavioural ecology. (b) A hypothetical workflow of behavioural ecologists and natural historians (green) working with molecular ecologists and bioinformaticians (blue), from project conception to completion (large grey arrow). Smaller arrows indicate each step with their width highlighting essential points in the collaboration. Note that DNA/RNA extraction and sequencing (shown as stippled) are likely to be outsourced.

Behavioural ecology was built on the foundation of Tinbergen's four questions (Tinbergen, 1963): we can only understand a behavioural trait by investigating its underlying mechanisms, how the trait develops, its evolutionary history, and its function (i.e. effect on fitness). Tinbergen stressed that answers to each question were complementary rather than mutually exclusive, and behavioural ecology has since grown into one of the most integrative fields in biological sciences (Monaghan, 2014). While genomic methods could become a useful part of our toolkit to answer aspects of each question, our goal is not to advocate for all behavioural ecologists to become experts in genomics. Neither should molecular ecologists necessarily all become experts in behaviour. Rather, we should form collaborative teams that make use of our wide range of complementary skill sets (Figure B1): the four specialists represent the different levels of inquiry from conceptual question framing to technical problem solving and move between phenotypic and genotypic approaches. Naturally, the number of people does not need to be four—many scientists may sit closer to the centre on both axes and thus bridge the gap between solely phenotypic or genotypic approaches.

To illustrate the potential benefits of interdisciplinary collaboration here we present a hypothetical workflow (Box 2, Figure B1b) from planning, data collection and analysis to reporting. The specific sampling design required for genetic samples, what phenotypes need to be quantified, and even the genomic analyses necessary to test hypotheses will require input from each team member: Behavioural ecologists ask *hypothesis‐driven* questions based on detailed knowledge of *the natural history of the study organism* to account for different selection pressures and confounding factors. However, jumping to the genomic era will require detailed knowledge of the *available methods and the theory* behind them to enable efficient communication between fields. Collecting *accurate behavioural data* and analysing it in a meaningful way, or even finding and sampling *enough individuals* in their natural habitats requires field skills. DNA extraction and sequencing can often be outsourced, yet the subsequent preprocessing of the massive genomic data and *choosing appropriate methods* to draw biologically meaningful inferences from it requires knowledge of *bioinformatics*. Finally, collaborative discussions throughout the process will aid the team in reporting their results and conclusions in a precise but understandable way to the benefit of wider audiences interested in behavioural evolution.

## 2 AVIAN BROOD PARASITISM AS A CASE STUDY

**FIGURE 2:**
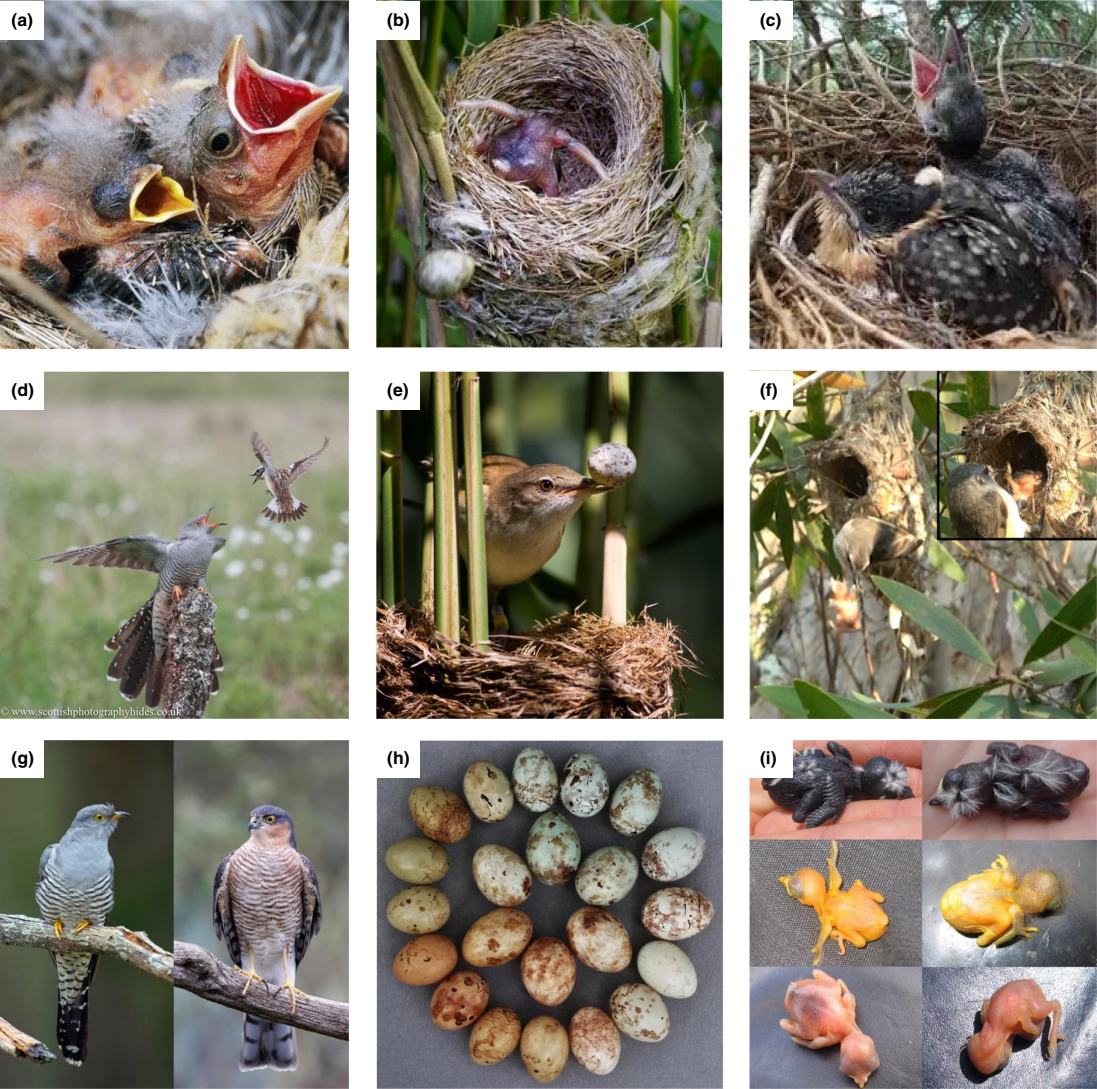
Examples of the arms races between avian brood parasites and their hosts. Row 1: Virulence (a) low—bronzed cowbird *Molothrus aeneus* with Bewick's wren *Thryomanes bewickii* host chicks, (b) high—common cuckoo removing reed warbler's egg (c) mutualism—great spotted cuckoo and crow *Corvus corone* host; Row 2: Defences (d) mobbing, (e) egg rejection, (f) chick rejection; Row 3: Mimicry (g) hawk mimicry by adult *Cuculus* cuckoos, (h) *Prinia* egg mimicry by cuckoo finch, (i) host chick (left) mimicry by *Chalcites* cuckoo species (right). Image credits: (a) Rolf Nussbaumer/Alamy Stock Photo, (b) Richard Nicoll, (c) Vittorio Baglione. (d) Alan McFadyen/Scottishphotographyhides.com, (e) Oldrich Mikulica. (f) Alfredo Attisano, (g) left panel: Frans Lemmens/Alamy Stock Photo; right panel, Mike Lane/Alamy Stock Photo, (h) Claire Spottiswoode, (i) Naomi Langmore.

Obligatory brood parasites (about 1% of birds, some insects and the cuckoo catfish, Thorogood et al., 2019) trick hosts into rearing foreign offspring as their own. The resulting selection for host defences, and counter‐adaptations in avian brood parasites (Figure 2), for example, has provided behavioural ecologists with a tractable system to study coevolution in the wild. Experimental methods to test hypotheses about behavioural defences and parasite trickery are well‐defined (Davies, 2011), and technological developments are widely used to quantify phenotypes (e.g. egg pattern and colour to identify individual cuckoos *Cuculus canorus*; Šulc et al., 2022; van den Berg et al., 2020). Compared to the rich body of knowledge describing behavioural defences and offence; however, almost nothing is known about the molecular mechanisms underpinning these traits, and heritability remains mostly assumed. The field of genetics has, however, already provided some insight into the evolutionary history of brood parasitism (Krüger & Pauli, 2017) and host‐race specialisation (Fossøy et al., 2011, 2016; Gibbs et al., 2000) and emerging work is starting to demonstrate the power of using genomic data to understand the genetic architecture of parasite adaptations (Merondun et al., 2024) and the evolutionary consequences of selection pressures exerted by hosts (Langmore et al., 2024). Nevertheless, the adoption of genomic tools remains limited to only a fraction of parasite and host species (e.g. there are at least 140 known host species of common cuckoos), and we are only starting to scratch the surface of what is possible (Brown et al., 2020; DaCosta & Sorenson, 2016; Lynch et al., 2020; Mills et al., 2020; Ruiz‐Rodríguez et al., 2018; Spottiswoode et al., 2022; Wang et al., 2016; discussed in relevant sections below).

Despite repeated predictions that genomics would re‐revolutionise the study of avian brood parasitism as molecular methods did in the 90s (Sorenson & Payne, 2002; Stoddard & Kilner, 2013; Tanaka, 2016), there has been relatively little work making use of data or insights from high‐throughput sequencing (Figure 1 and Figure S1). This is surprising when compared to the use of genomics to tackle other evolutionary questions about hosts and parasites (Andrews et al., 2016) and the contributions of biologists working on avian genetics and genomics (Wink, 2019). For example, they were instrumental in developing the field of quantitative genetics with an animal model approach (Merilä & Sheldon, 2001), generated a detailed understanding of avian genome structure (Zhang et al., 2014) and conducted some of the first studies of genomic adaptation in wild populations (Bay et al., 2018; Kraus, 2019). Therefore, here we discuss how applying genomic tools to avian brood parasitic systems could be used to better predict (i) where and when brood parasitism should evolve, (ii) when and how hosts defend, or (iii) how coevolutionary trajectories depend on ecological change, three major questions in a field aiming for a deeper understanding of coevolutionary dynamics (Thorogood et al., 2019). Even though we focus on long‐standing questions in avian brood parasitism, these three key concepts (evolutionary origins, plasticity, geographic selection mosaics) are of broad relevance across ecology and evolution.

### 2.1 Where and when should brood parasitism evolve?

Darwin (1859) proposed that avian brood parasitism evolved from an ancestor with parental care. Since then, three key pathways have been suggested (see Krüger & Pauli, 2017 for an in‐depth discussion): by evolving directly from parental care (Yom‐Tov & Geffen, 2006), via conspecific brood parasitism as a ‘stepping stone’ (Lyon & Eadie, 1991; Payne, 1977), or via cooperative breeding as a precursor (Hamilton & Orians, 1965). Ecological conditions and changes in life‐history traits are also likely to have influenced the transition (Davies, 2000; Hamilton & Orians, 1965). However, previous attempts to test these hypotheses using phylogenetic comparative methods have produced mixed results depending on the phylogeny used. For example, the first analyses to use molecular phylogenies developed in the early 2000s (Aragón & Soler, 1999 from cyt*b*, Johnson et al., 2000 from cyt*b*, and ND2 mitochondrial genes) suggested that parasitism evolved in Cuculidae cuckoos to reduce the costs of reproduction associated with migration and a change in diet (Krüger & Davies, 2002) while a similar analysis using an improved phylogeny with a different topology and branch lengths suggested parasitism evolved *before* migration (Boerner & Krüger, 2008). Even these results may not be accurate, however, as the phylogeny they used included only a small fraction of the mitogenome and no nuclear markers (Sorenson & Payne, 2005), leaving some of the older branching events and relationships among subfamilies unresolved. This problem is retained in the most up‐to‐date phylogenies of birds whether they prioritise taxonomic coverage (Jetz et al., 2012) or use more markers (Prum et al., 2015). Furthermore, brood parasitism has evolved independently only seven times in birds, which has limited the statistical power of previous comparisons. How can genomic tools be used to resolve fundamental questions about the origins of brood parasitism?

The first step would be to use genomic data to improve the resolution of a phylogeny that includes as many extant brood parasites as possible, as well as their hypothesised relatives. While this could be achieved using reduced sampling methods e.g. ultraconserved elements (UCEs) or RAD‐seq (Manthey et al., 2016), whole‐genome data becoming available from e.g. the Bird 10,000 Genome (B10K) project (Feng et al., 2020) would improve phylogeny robustness and provide more in‐depth information to estimate evolutionary pathways and trait evolution (Kapli et al., 2020). For example, incongruences between species and single‐gene trees can be used to detect rapid speciation and diversification events associated with life‐history changes (e.g. via incomplete lineage sorting and/or introgression; Cole et al., 2022). Or, whole‐genome sequences from multiple parasitic and non‐parasitic species could be compared in a phylogenetic context to detect e.g. gene loss (Feng et al., 2021; Zhang et al., 2014) related to parental care, and/or molecular adaptations possibly associated with a transition to parasitism (e.g. positively selected genes, rapidly evolving genes, pseudogenes; Cole et al., 2022). This approach would also enhance predictions of both the timing and the ecological and biogeographical conditions that facilitate the evolution of this strategy (e.g. see Cole et al., 2022 for an example investigating behavioural transitions in penguins). Moving from phylogenetics to comparative phylogenomics however requires careful consideration of the potential pitfalls in big data statistics (Cornuault & Sanmartín, 2022; Young & Gillung, 2020) and model interpretation (Louca & Pennell, 2020).

Genomic approaches can also bypass the limitation of seven independent origins of avian brood parasitism, since comparing whole genome or transcriptomic data of even a single brood parasitic species with non‐parasitic relatives can reveal interesting evolutionary pathways. For example, comparing genome structure and gene expression among just three species (representing a kinetoplastid host, a protozoan parasite, and its closest known free‐living relative) enabled Jackson et al. (2016) to pinpoint patterns of genomic streamlining (i.e. gene loss for functional redundancy and subsequent radiations of novel gene families) associated with becoming a parasite. Transcriptomics has also started to be used to explore the loss of parental care in avian brood parasites. By comparing differentially‐expressed genes in the preoptic area of the brain (implicated in parental behaviour across vertebrates) with three related Icterid species (using RNA‐seq data), Lynch et al. (2019) suggested a molecular pathway for the loss of parental care as the two parasitic species showed more juvenile‐like (i.e. neotenic) patterns of gene expression than their non‐parasitic sister species. Phenotypes are now thought to be more likely to arise from changes in gene interactions than novel gene mutations (Heng, 2019), so investigating differential gene expression at the network level (e.g. gene co‐expression network alignment, Ovens et al., 2021) is likely to be a particularly helpful next step in inferring when and how brood parasitism evolved. Furthermore, while interpreting the evolutionary significance of comparative patterns of gene expression (or any genomic data) is challenging without knowledge of gene function, gene networks can also help to infer function (Ovens et al., 2021; Raina et al., 2023). The specific functions and regulatory pathways of genes associated with behavioural traits in higher organisms remain poorly known (Box 1), but as more functional annotations of genes involved in parental care become available, it may become possible to compare between different parasite–host systems and track the evolution of brood parasitism back in time.

Comparative analyses like these described above are more reliable and powerful when conducted with multiple species across a well‐resolved phylogeny (Deutekom et al., 2019). However, even if the phylogeny for brood parasites is improved using genomic data, analyses may still be limited by a lack of behavioural and ecological data. For example, a phylogenetically controlled study investigating associations between brood parasitism and the characteristics of Australasian and sub‐Saharan African species used as hosts (Feeney et al., 2013) suggested that brood parasitism promotes the evolution of cooperative breeding (e.g. for improved host defences) but a second study with a dataset expanded to include hosts of brood parasites from Asia and the Americas concluded the opposite—brood parasites are attracted to cooperatively breeding hosts for enhanced parental care (Wells & Keith Barker, 2017). In common with many studies of behaviour (Brouwer & Griffith, 2019; Cockburn, 2020), ecological data for brood parasites has traditionally been highly biased towards temperate regions (Thorogood et al., 2019), leaving replicated data on reproductive mode and other life history traits of brood parasite (and host) taxa in the tropics lagging behind. Although this is improving (see Kennerley et al., 2022), it is likely that targeted sampling of brood parasites and families taking both phylogenetic and biogeographical bias into account (Brouwer & Griffith, 2019) will be necessary to finally determine when, why and how brood parasitism has evolved in birds.

### 2.2 When and how should hosts defend?

Explaining when and *how* hosts evolve defences is a key question in any host–parasite system as it determines the persistence of both. However, while brood parasite hosts have provided excellent examples of behavioural defences (Figure 2), we still lack a comprehensive understanding of how defences arise, why they sometimes persist in allopatry (including during speciation events, e.g. Kuehn et al., 2014), or whether the absence of resistance in some hosts indicates evolutionary lag (i.e. there has not yet been time to evolve defences) versus alternative strategies to cope with the fitness costs of parasitism (i.e. tolerance, Avilés, 2017). One model suggests that behavioural plasticity might provide an answer (Manuel Soler, 2014), where changing environments and selection pressures lead species to either lose plasticity (e.g. 100% of individuals reject foreign eggs, cuckoo goes extinct), or lose the trait (i.e. host goes extinct). However, this model is challenging to test since the absence of a behavioural response in the field does not necessarily mean that the ability to perform the defence is absent (i.e. genetic polymorphism vs. behavioural plasticity). For example, hosts may ‘fine‐tune’ costly egg rejection defences to match their perceived risk of parasitisim (Thorogood & Davies, 2013) and even in populations or species that are allopatric from parasites, altering the perceptual (Lahti, 2006) or physical (Yang et al., 2020) challenge of identifying cuckoo eggs experimentally can elicit apparently ‘lost’ egg rejection. However, in‐depth knowledge about plasticity and/or tolerance is unfortunately still lacking beyond a handful of host species (Cotter et al., 2019; Soler, 2014). How could we use genomic data to disentangle hypotheses for trait absence and determine when and how defences evolve?

Field methods to test for plasticity and tolerance require careful (and time‐consuming) design to rule out cryptic defence traits and it is not feasible to test for plasticity in every current, potential or past host species. If we knew the genetic basis for a defence behaviour then it would be possible to test hypotheses about evolutionary histories across populations or species lacking such high‐quality behavioural data. However, attempts to ‘find the gene’ for egg rejection by comparing individuals within species have had limited success: while using microsatellites to estimate levels of gene flow between parasitised and unparasitised magpie *Pica pica* populations (Martín‐Gálvez et al., 2006, 2007) helped to identify a candidate marker for its rejection of great spotted cuckoo *Clamator glandarius* eggs (Martín‐Gálvez et al., 2006), the same marker was not associated with egg rejection in a host species of common cuckoos (the great reed warbler *Acrocephalus arundinaceus*, Procházka et al., 2014). This may be because both host species adjust their defences based on experience and context (Martínez et al., 2020; Moskát et al., 2014), making detection of genetic associations with the ‘absence’ of the trait challenging, and this line of inquiry has not continued into the genomic era.

Instead, we could use a combination of comparative genomic analyses to determine which species would be best to target next with limited resources for field experiments. Rather than rely on genome‐wide association studies within species (best suited for detecting large‐effect genes, see Box 1), genome architectures could be compared across host species. High‐quality reference genomes are now available for brood parasite hosts with rich behavioural data (e.g. superb fairy wren *Malurus cyaneus*: Peñalba et al., 2019, great reed warbler: Sigeman et al., 2020; Westerdahl et al., 2022, common reed warbler *Acrocephalus scirpaceus*: Sætre et al., 2021) and comparing chromosome synteny among groups of well‐studied host species that show little or no plasticity in their defences (e.g. some past and present cowbird hosts, Kuehn et al., 2014; Rothstein, 2001) could reveal e.g. candidate inversions associated with behavioural differences (see Bentley et al., 2022 for a recent example of using this approach to detect evolution of sensory and immune system adaptations with sea turtles). Identifying the nature of the genomic regions underlying defences would also greatly improve our understanding of trait evolution (Spottiswoode et al., 2022). For example, if egg rejection behaviour is associated with an inversion (detected using comparative genomics), then it may be difficult to evolve as it requires large changes in the genome. However, it could arise via introgression from other populations (see Question 3) or during speciation (Stolle et al., 2022). If similar changes to genomic regions were shared among host species, results could be used to develop markers to detect defences in species where either behavioural data is unknown or difficult to obtain, or where they may be cryptic/plastic. Broad‐interest research efforts are rapidly increasing the availability of reference genomes (e.g. Vertebrate Genomes Project, Paez et al., 2022; B10K, Feng et al., 2021) to complement the ones already available for host species where defences have been tested (Peñalba et al., 2019; Sætre et al., 2021; Sigeman et al., 2020; Westerdahl et al., 2022), but even using improved genome‐based phylogenies for birds (Feng et al., 2021) could help in targeting research efforts. For example, a species that never expresses a defence although all of its close relatives do, could be interesting to target for high‐quality genome sequencing and behavioural experiments designed to induce plasticity of host defences.

The second genomic approach that can be taken without prior knowledge of the genes underlying behavioural defences would be to use population genetic theory within species to ask if observed genomic patterns fit one of the evolutionary scenarios (i.e. missing adaptation vs. tolerance). In practice, this would require combining multiple approaches to account for different evolutionary forces causing genomic patterns. For example, if we have both behavioural and genomic data from host populations before and after invasion by cuckoos, we could use simulations to compare allele frequency changes to a neutral model without selection (i.e. using coalescent theory, Beichman et al., 2018). Such changes can then be compared to the output of flexible simulation frameworks (SLiM, Haller & Messer, 2019; Nemo, Guillaume & Rougemont, 2006), which take key parameters inferred from the populations of interest (such as mutation rate and fitness) into account to test different evolutionary processes. Although it is clear that rejecting a virulent parasite will improve a host's reproductive success, the heritability of expressing a defence (or not) is yet to be estimated for any brood parasite–host but is necessary to test evolutionary models accurately. To overcome this problem, we could sequence (behaviourally tested) acceptor and rejector individuals from a recently parasitised population (where selection for rejection is strong) to build a pedigree from the genomic data and quantify fitness based on reproductive output (Chen et al., 2019). This approach could be combined with others focusing on nascent selective sweeps when detecting genomic regions with longer runs of homozygosity (Gautier et al., 2017). Finally, genomic estimates of effective population size through time could also inform the fitness trajectory of various populations before and after invasion and be combined with evolutionary simulations (Mathur et al., 2023). Study systems sampled in at least two‐time points with a known parasitism history can be difficult to find in the wild, although ongoing range shifts due to climate change, habitat modification, or invasions could provide amenable study systems (see Grim & Stokke, 2016).

### 2.3 Will coevolution persist across time and space with ecological change?

**FIGURE 3:**
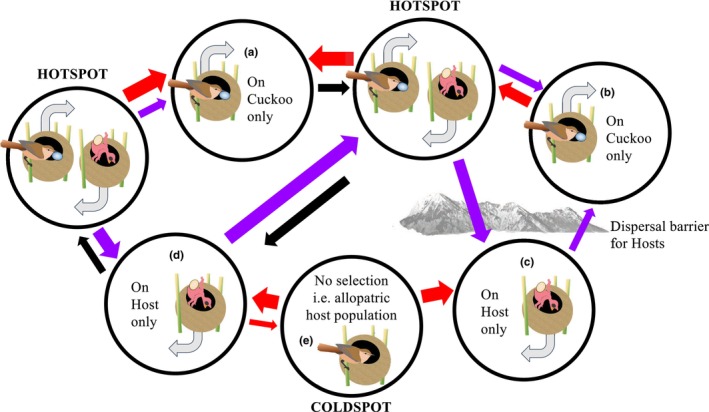
A hypothetical geographic mosaic of coevolution between a brood parasitic cuckoo and a warbler host (key components in bold text), modified from (Thompson, 2005): Circles represent locations where **selection varies** and arrows between circles describe the direction of **gene flow** (red is host, purple is cuckoo, and black represents similar gene flow of both). Arrow thickness indicates **trait remixing** essential for maintaining genetic variation: If gene flow is absent then local fixation of alleles increases, whereas high levels of gene flow cancel out local adaptation. Reciprocal selection occurs in ‘hotspots’ and non‐reciprocal selection occurs in ‘coldspots’; these vary according to local population dynamics e.g. (a) strong gene flow of experienced hosts or (b) locally fixed host defences exert stronger selection on local cuckoos, or (c) a recently invaded host population lacks defences or (d) ‘spill over’ of cuckoos from another host species exerts stronger selection on hosts. Coldspots also arise when (e) hosts and cuckoos do not co‐occur (i.e. relaxed selection). Behaviour could influence each component: Local environmental conditions determine the relative fitness of expressing or retaining behavioural defences (especially when plastic) when parasitism is low (i.e. strength of selection), movement of behavioural phenotypes is unlikely to be homogenous (i.e. affecting direction and specific genotypes during gene flow), and both defences and population dynamics can depend on the behaviour of others' phenotypes/genotypes (i.e. indirect genetic effects).

As discussed in Question 2, all potential host species should in theory evolve defences and render parasitism untenable given sufficient time (also see Rothstein, 1990). How is it possible then, that brood parasites have persisted for millions of years (Question 1)? Furthermore, and perhaps most importantly, can we predict what will happen next to hosts and brood parasites, given rapid environmental change? The Geographic mosaic of coevolution theory (GMT, Thompson, 1999, 2005) provides a compelling framework to answer these questions. First, it explains how antagonistic coevolution can continue for long periods: local environmental variation, population dynamics and demographics, gene flow, mutation and drift combine to produce mosaics of reciprocal selection (‘hot spots’) and non‐reciprocal selection (‘cold spots’) in time and space (Figure 3). The key cold spot that allows parasites to persist is where selection on hosts is sufficiently relaxed that they lose their defences. This then allows parasites to eventually reinvade (although some work suggests that it is variation in the parasite's virulence that determines long‐term success (Kaur et al., 2019). Second, insight into the component parts of GMT can better facilitate predictions about adaptive potential and interacting species' resilience to rapid environmental change (Benkman et al., 2008; Hoberg & Brooks, 2015; Penczykowski et al., 2016; Thorogood et al., 2020). However, there have been few attempts to test GMT with avian brood parasites and their hosts (Ruiz‐Raya & Soler, 2017), despite them being a putative example in the theory's seminal publications (Thompson, 1999, 2005). Behavioural experiments have revealed spatial correlations between host and parasite traits (e.g. reed warblers vs. common cuckoo: Davies & Brooke, 1988; Lindholm, 1999; Lindholm & Thomas, 2000; Stokke et al., 2008; magpies vs. great spotted cuckoo: Soler et al., 1999); prinias and parrotbills vs. common cuckoo: Yang et al., 2015, 2020), providing evidence for hot spots as well as cold spots where parasitism is absent and defences vary, but we lack quantitative estimates of the strength of selection and trait remixing at the genomic level. These estimates are essential (Nash et al., 2008; Nuismer et al., 2010) to explain how coevolutionary interactions persist in time (Gomulkiewicz et al., 2007). There have been some attempts to quantify gene flow and local allele frequencies of a putative candidate marker for egg rejection in magpies (Martín‐Gálvez et al., 2007), but this is where studies on brood parasitism, and tests of GMT for behavioural coevolution more broadly, have hit a stumbling block.

How can we quantify selection mosaics and trait remixing in avian brood parasite–host systems, given that the heritable mechanisms of coevolved traits remain largely unknown? Here again, combining empirical data with new molecular methods could offer ways forward. Landscape genomics (Gene–Environment Association studies, GEA, Table 1) uses genomic and environmental data collected across a species distribution, or along an environmental gradient of interest. Neutral markers are used to infer the underlying spatial and genetic population structure, environmental associations can be tested by incorporating e.g. abiotic climatic variables, and any remaining markers associated with the environmental variable of interest (e.g. current parasitism rate) are evidence of selection. However, this method has rarely been used to test for associations with biotic variables (Zueva et al., 2018). The space‐for‐time substitution inherent to landscape genomics (Rellstab et al., 2015) could also help solve a major issue: detecting selection usually requires long‐term data (Teplitsky et al., 2014). However, behavioural studies are rarely replicated in time and space, although recent invasions of brood parasites to new areas and range edge populations provide unique opportunities to observe coevolution in action (Grim & Stokke, 2016). Studying spatial variation in egg polymorphism (Yang et al., 2017) and egg rejection (Grim & Stokke, 2016) has been suggested to overcome the lack of long‐term behavioural datasets, but there have thus far been no attempts to evaluate the genomic consequences of environmentally varying brood parasitism risk in avian hosts.

With these landscape and comparative genomic approaches, it becomes possible to directly measure levels of selection, gene flow and trait remixing. A major strength of genomic approaches is the possibility to apply several methods with the same dataset to strengthen results: GEA can be combined with QTL (Quantitative Trait Locus analyses, Table 1) or GWAS (Genome‐Wide Association Studies, Table 1) to further study which loci are associated with host–parasite coevolution and which with unrelated environmental factors. In systems where we have detailed knowledge of host and parasite behaviour, demography, and genomics, it could therefore be possible to move beyond the phenotypic gambit. For example, genome‐wide markers have been associated with climate adaptation in yellow warblers *Setophaga petechia*, and used to predict future vulnerability to climate change (Bay et al., 2018). Yellow warblers, however, are also a common host of the brown‐headed cowbird *Molothus ater* and the expression of host defences and levels of parasitism vary geographically (Kuehn et al., 2016). These data could be used to zoom in and look for the molecular mechanisms underlying behavioural coevolutionary adaptations (Box 1). Molecular resources are also becoming available for the reed warbler (Sætre et al., 2021), in addition to the wealth of existing knowledge on geographic variation in defence behaviours (Stokke et al., 2008). Studying such avian brood parasites and host systems would complement the current interest in detecting molecular evidence for biotic selection as examples of behavioural coevolution are few (e.g. ants: Kaur et al., 2019), and most studies of the genomic changes associated with defense traits in antagonistic coevolution come from systems in vivo (Nair et al., 2019). However, accurate predictions of future coevolutionary trajectories require further development of models that take all evolutionary forces into account, and not only selection (i.e. effective migration, mutation, recombination, and drift, Hahn, 2018; Luikart et al., 2018). This is a major goal in population genomics, and methods are rapidly developing to e.g. better understand the ecological processes that affect coevolutionary dynamics (Amandine et al., 2022) and to trace back the geographical locations of genetic ancestors (Osmond & Coop, 2021). In summary, analysing genomic data along with behavioural data in a brood parasite–host system in the wild could not only test the theory of a geographic mosaic of coevolution using direct genetic evidence but also help study three avenues of inquiry: speciation of hosts and parasites, persistence of antagonistic coevolutionary interactions in time and maintaining resilience to rapid ecological change.

## 3 CONCLUDING REMARKS

Here we have shown that investing in analysing genomic data along with behavioural data in a brood parasite–host system could lead to advances in understanding the evolutionary origins of behavioural strategies, the fitness outcomes of plastic trait expression, the persistence of antagonistic coevolutionary interactions in time, and how resilience to rapid ecological change may be maintained. These themes are of broad interest beyond avian brood parasitism and show that genomic tools can be used to find answers to more than mechanistic questions. By applying genomic comparisons at different levels ranging from within individuals to between populations and species, we can also address how behaviour develops or changes through ontogeny, its adaptive value, and its evolutionary past. In other words, genomic tools can be integrated with behaviour to find answers to all of Tinbergen's ‘four questions’ (see Box 2/Table 1 and outstanding questions in Box 3).

BOX 3 **Outstanding questions**

- Do rates of molecular and phenotypic evolution vary between avian brood parasites and hosts?
- Egg coloration has long been a focus but we have not discussed it here. What are the molecular mechanisms that facilitate rapid changes in egg colouration during evolving host–parasite arms races? How is egg or plumage colouration polymorphism maintained?
- What platforms are needed to best bring behavioural ecologists and molecular ecologists together to better integrate Tinbergens' four questions?
- If we find candidate genes for behaviours, can field‐friendly methods be developed to experimentally test gene function?
- When does behavioural plasticity facilitate or hinder adaptation? This will require a deeper integration of behavioural experiments with epigenomics.
- Can a richer understanding of behavioural interactions improve heritability estimates in studies of genetic and non‐genetic inheritance (or indirect genetic effects)?
- Do new behavioural adaptations occur from selection acting on standing genetic variation, introgression or new mutations?

Although we have emphasised in our review what can be done with genomic tools beyond finding the gene for behaviour, it is important to note that genomic and phenotypic data, and insights about function, evolutionary history, and plasticity gained from these approaches can also complement a mechanistic understanding of trait heritability (Box 1). For example, comparative analyses among species (e.g. phylogenetic comparative method, Question 1; chromosome synteny, Question 2) may identify a genomic region that appears repeatedly in association with a behavioural trait. Or, using landscape genomic approaches that compare across space rather than time (e.g. Question 3), regions associated with current selection would be good candidates for more mechanistic approaches. Or, vice versa, if we knew the ‘gene for behaviour’, we could for example track the evolutionary history of the behaviour more precisely across taxa (Question 1), determine the capacity for a behaviour directly from the genotype without behavioural testing (i.e. ‘reverse ecology’, Li et al., 2008), disentangle the heritable and plastic components of trait expression (Question 2), collect more direct evidence of selection (Questions 2 & 3), and even predict future adaptations (Kaur et al., 2019). Indeed, our review is not intended to negate the need for behavioural genomics but if we can use broad approaches across teams, we may be more likely to come closer to the key goal of understanding trait evolution.

Throughout this article, we have argued why integrating genomics into behavioural ecology could be beneficial, but behavioural ecologists could also help resolve several outstanding issues in genomics. For example, genomics has been criticised for being data‐driven rather than led by hypotheses (Kell & Oliver, 2004; Stern, 2019), and increasingly reliant on searching for correlations rather than experiments to test causation (Voit, 2019). At the same time, adopting a Tinbergian approach from behavioural ecology could help move forwards as it integrates mechanisms with development, function and evolutionary history to test hypotheses (see Nesse, 2013 for discussion of how this could revolutionise many fields). Similarly, variation in behaviour is often perceived to be more difficult to measure than physical traits, but the underlying heritability of both is intertwined (Auman & Chipman, 2017; Davidson, 2010). Reading genome sequences has been insufficient to explain phenotypic variation (Battaglia, 2020; Charney, 2012; Chevin et al., 2022) and has instead revealed that closing the gap requires understanding links between the genotype, phenotype, environment and species interactions across biological levels (Corning, 2020; Sanger & Rajakumar, 2019). Behavioural ecologists could bring a deep understanding of how and what to measure, as well as the ecology underlying the trait in question (Vignal & Eory, 2019), to frame hypotheses appropriately. Since genomics are evolving rapidly, any step‐by‐step guide for integrating genomic tools to behavioural ecology will soon be outdated. Instead, we urge forming collaborative teams (Box 2) to make use of our wide range of complementary skill sets. We now have a plethora of sequencing techniques and massive datasets becoming available, and utilising these data will require appropriate analysis methods that are carefully designed to address questions about polygenic traits with inherent plasticity. Working together we can finally close the phenotype–genotype gap (Kratochwil & Meyer, 2015).

## AUTHOR CONTRIBUTIONS

**Katja Rönkä:** Conceptualization (equal); data curation (lead); formal analysis (lead); funding acquisition (equal); investigation (lead); methodology (lead); project administration (lead); resources (equal); validation (equal); visualization (equal); writing – original draft (lead); writing – review and editing (equal). **Fabrice Eroukhmanoff:** Investigation (supporting); writing – original draft (supporting); writing – review and editing (supporting). **Jonna Kulmuni:** Investigation (supporting); visualization (supporting); writing – original draft (supporting); writing – review and editing (supporting). **Pierre Nouhaud:** Investigation (supporting); visualization (supporting); writing – original draft (supporting); writing – review and editing (supporting). **Rose Thorogood:** Conceptualization (equal); data curation (supporting); formal analysis (supporting); funding acquisition (equal); investigation (supporting); methodology (supporting); project administration (supporting); resources (equal); supervision (lead); validation (equal); visualization (equal); writing – original draft (supporting); writing – review and editing (equal).

## FUNDING INFORMATION

This work was supported by HiLIFE Helsinki Institute of Life Sciences [starting grant to R.T., fellows grant to J.K.], the Academy of Finland [grant number 1333803 to R.T., 309580 to J.K., 1347478 to K.R.] and the Ella and Georg Ehrnrooth Foundation [grant to K.R.].

## CONFLICT OF INTEREST STATEMENT

The authors have no conflicts of interest to declare.

## Supporting information


Figure S1.

Table S1.

## Data Availability

ISBE conference abstracts books were obtained by R.T. from the congress organisers. Titles of the ISBE abstracts including genomic methods are listed in the Table S1.
