# Supplementary material for: Beyond genes‐for‐behaviour: The potential for genomics to resolve long‐standing questions in avian brood parasitism
Source: Ecol Evol. 2024 Nov 17;14(11):e70335. doi: 10.1002/ece3.70335 (PMC11581780; doi:10.1002/ece3.70335)
Supplement: Supplementary file 1 — Figure S1. [file ECE3-14-e70335-s002.pdf]

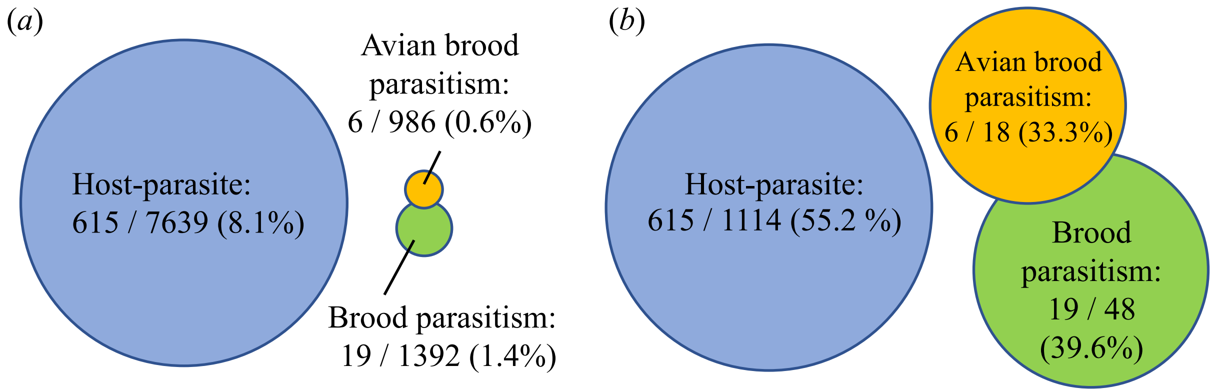

Supplementary Figure 1. Proportions of studies published on host-parasite interactions (blue), brood parasitism (green, regardless of taxa) and avian brood parasitism (yellow) using genomics (i.e. using high-throughput sequencing techniques) relative to (*a*) all studies and (*b*) to studies using molecular methods. While genomics studies are few in brood parasitism (*a*), when molecular methods are used then a similar proportion use genomics (*b*). Results come from a Scopus search limited between 1.01.2006 and 5.10.2022 (high-throughput sequencing became available in 2005). **Search details**. Molecular methods articles were searched in Scopus using the keywords dna OR rna and excluding articles using “genomics”. By “genomic” tools we refer to next-generation and third generation methods using high-throughput sequencing, which were searched with the following keywords: “next generation sequencing”, “high throughput”, “deep sequencing”, “transcriptom*”, “*omics*”, “RNAseq*”, “RNA-seq*”, “RNA -seq*”, “RNA seq*”, “RNA - seq*”, “RADseq*”, “*RAD-seq*”, “*RAD -seq*”, “*RAD seq*”, “*RAD - seq*”. Brood parasitic studies were searched with the key word “brood parasit*” and host-parasite with “host parasit*” (excluding the brood parasit* search results). Studies on obligatory avian brood parasites were separated from other brood parasite studies by exluding search results by the following keywords: “intraspecific”, “conspecific”, "insect", "Hymenopt*", "Lepidopt*", "Apoidea", "Hexapoda", "Coleopt*", "beetle", "butterfly", "ant", "wasp", "honeybee", "bee", "bees", "aphid", "oviposition", "fish". In all searches documents corresponding with “cuckoo search” (an inconveniently named algorithm) were excluded and the types of search documents limited to journal articles.
